# Functional Trajectories and Quality of Life in Post-Acute Skilled Nursing Facility care after hospitalization

**DOI:** 10.1093/geroni/igab046.3001

**Published:** 2021-12-17

**Authors:** Sandra Shi, Brianne Olivieri-Mui, Ellen McCarthy, Dae Hyun Kim

**Affiliations:** 1 Hebrew SeniorLife, Harvard Medical School, Roslindale, Massachusetts, United States; 2 Hebrew SeniorLife, roslindale, Massachusetts, United States

## Abstract

Frailty predicts readmissions and mortality after acute hospitalizations. Understanding whether frailty predicts functional recovery after acute hospitalizations may help guide post-acute care and rehabilitation. This feasibility study enrolled 24 adults aged ≥65 years from a skilled nursing facility (SNF) after acute hospitalization. We calculated a deficit-accumulation frailty index (FI range: 0-1; non-frail [≤0.25], mild frailty [0.26-0.35], moderate [0.36-0.45], and severe [>0.45]) via in-person assessment on SNF admission. We measured weekly functional improvement with modified Barthel Index, as well as quality of life. Modified Barthel Index and quality of life were measured weekly by Patient-Reported Outcome Measurement Information System (PROMIS) (standardized score with mean 50 and SD 10, higher is better). The mean age was 83.3 years [SD 8.0], and 17 (71.8%) were female. Length of stay for those with severe frailty (FI>0.45) was 26.8 days [10.7] compared to those who were not frail, mildly frail, or moderately frail (13.3 [7.3], 9.4 [4.4], and 15.2 [4.9] respectively). Those with severe frailty also had delayed functional improvement (mean Barthel Index 48.6, 53.4, and 56.6 on admission, week 1, and week 2 of SNF admission respectively), compared to those with moderate frailty (mean Barthel Index 47.5, 69, 73) or mild frailty (68.3, 86, 90.5). Self-reported mental and physical health-related quality of life was relatively unchanged across SNF episode for all frailty categories. These findings suggest that older adults with moderate or severe frailty may experience a typical course of delayed functional recovery and that further monitoring may be necessary for prognostication.

